# Supplementary material for: Are Differences in Inflammatory Markers between Patients with and without Hypertension-Mediated Organ Damage Influenced by Circadian Blood Pressure Abnormalities?
Source: J Clin Med. 2022 Feb 25;11(5):1252. doi: 10.3390/jcm11051252 (PMC8911066; doi:10.3390/jcm11051252)
Supplement: Supplementary file 1 [file jcm-11-01252-s001.zip › Table S1.pdf]

Table S1. General features and 24-hour ABPM parameters of patients with and without hypertension-mediated organ damage (extended).

Variables	All patients n= 522	Non-HMOD n= 311	HMOD n= 211
Age (years)†	54±15	49±14	62±12[1]
Sex (female)‡	247(47)	169(54)	78(37) [1]
WC (cm)†	100±13	98±13	104±11[1]
BMI (Kg/m²)†	31±12	30±11	32±13
HR (bpm)†	71±13	72±13	70±12[1]
Current/Former smokers‡	153(29)	82(26)	71(33) [1]
Alcohol intake‡	161(30)	94(30)	67(31)
Non-dipper profile‡	232(45)	112(37)	120(57)[1]
HLP‡	365(69)	183(58)	182(86)[1]
DM‡	154(29)	60(19)	94(44) [1]
Office SBP (mmHg)†	144±18	141±16	149±19[1]
Office DBP (mmHg)†	84±11	84±11	83±11
24-hSBP (mmHg)†	128±13	127±11	130±14
24-hDBP (mmHg)†	76±10	78±10	74±11[1]
dSBP (mmHg)†	133±13	132±12	134±15
nSBP (mmHg)†	119±14	117±13	122±15
dDBP (mmHg)†	81±11	82±10	78±12[1]
nDBP (mmHg)†	68±9	69±9	67±10
SBPF (%)	10±7	11±6	8.5±7[1]
DBPF (%)	15±8	16±7	13±8[1]
Antihypertensive drugs‡	356(68)	176(56)	180(85)[1]
RAAS blockers‡	259(49)	117(37)	142(67)[1]
ACEI‡	30(6)	21(7)	9(4)
ARBs‡	225(43)	93(29)	132(62)[1]
Anti-aldosterone agents‡	14(2.7)	7(2.3)	7(3.3)
Diuretics‡	194(37)	81(26)	113(53)[1]
CCBs‡	73(23)	98(46)	171(32)[1]
B-blockers‡	56(10)	22(7)	34(16)[1]
Statins‡	174(34)	76(24)	98(46)[1]
Compliant patients‡/§	338(94)	171(97)	167(92)

HMOD–Hypertension-mediated organ damage. WC–Waist circumference. DM–Diabetes mellitus. SBP–Systolic blood pressure. DBP–Diastolic blood pressure. 24-hSBP–Average SBP over 24 hours. 24-hDBP–Average diastolic BP over 24 hours. dSBP–Average SBP during the day. nSBP–Average SBP during the night. dDBP–Average DBP during the day. nDBP–Average DBP during the night. SBPF–SBP fall. DBPF–DBP fall. RAAS–Renin-angiotensin-aldosterone system. ACEI– Angiotensin converting enzyme inhibitors. ARBs–Angiotensin II receptor blockers. CCBs–Calcium channel blockers. Results expressed as † refer to mean ± standard deviation, ‡ refer to number (%) and § refer to n= 356 patients.

[1]Indicated comparison with patients without HMOD ($P < 0.05$)
