# Supplementary material for: Are Differences in Inflammatory Markers between Patients with and without Hypertension-Mediated Organ Damage Influenced by Circadian Blood Pressure Abnormalities?
Source: J Clin Med. 2022 Feb 25;11(5):1252. doi: 10.3390/jcm11051252 (PMC8911066; doi:10.3390/jcm11051252)
Supplement: Supplementary file 1 [file jcm-11-01252-s001.zip › Table S2.pdf]

Table S2. Differences in albumin: creatinine ratio, carotid intima-media thickness, ankle-brachial index and left ventricular hypertrophy between patients with and without HMOD.

**Variables**	**All patients**	**Non-HMOD**	**HMOD**
ACR(mg/g)†	12±72	2.03±4.5	22.1±10[1]
Carotid IMT(mm)†	0.80±0.15	0.72±0.09	0.89±0.16[1]
ABI†	1.14±0.16	1.16±0.12	1.12±0.20[1]
LVH n (%)‡§	181(43)	7(3)	48(26)[1]

HMOD–Hypertension-mediated organ damage. ACR–Albumin: creatinine ratio. IMT–Intima-media Thickness. ABI–Ankle-brachial index. LVH–Left ventricle hypertrophy. Results expressed as † refer to mean ± standard deviation and ‡ refer to number and percentage. § refers to n= 422 patients.

[1]Indicated comparison with patients without HMOD ($P < 0.05$)
