# Supplementary material for: Are Differences in Inflammatory Markers between Patients with and without Hypertension-Mediated Organ Damage Influenced by Circadian Blood Pressure Abnormalities?
Source: J Clin Med. 2022 Feb 25;11(5):1252. doi: 10.3390/jcm11051252 (PMC8911066; doi:10.3390/jcm11051252)
Supplement: Supplementary file 1 [file jcm-11-01252-s001.zip › Table S3.pdf]

Table S3. Multivariate logistic regression analysis for HMOD with and without including the circadian blood pressure profile.

**Variables**	***P*-value**	**Odds ratio (OR) for HMOD**	**CI 95%**	
Age (> 50 years)	<0.001	3.628	2.078	6.337
Sex (female)	.052	.577	.331	1.005
Smoking status	.216	1.462	.802	2.665
Alcohol intake	.188	.678	.380	1.209
WC (>98 cm)	.346	1.276	.769	2.118
Non-dipper profile	.171	1.387	.868	2.215
DM	.072	1.656	.956	2.866
HLP	.206	1.473	.808	2.684
ARBs	.021	2.133	1.120	4.062
CCBs	.749	.904	.488	1.675
Diuretics	.924	.968	.493	1.900
Statins	.855	.949	.545	1.655
PTC (<275 10^3/µl)	.011	2.010	1.176	3.434
ESR (>20 mm/h)	.044	1.775	1.015	3.103
Fibrinogen (>320 mg/dL)	.001	2.882	1.573	5.277
Uric acid (>7 mg/dL)	.048	1.806	1.005	3.248
Ferritin (>150 µg/L)	.486	1.184	.736	1.904

Omnibus test for coefficients (Chi^2): $P< 0.05$. Cox and Snell R-squared: 0.281. Nagelkerke's R-squared: 0.378. Sensitivity= 0.72. Specificity= 0.79.

WC–Waist circumference. ESR–Erythrocyte sedimentation rate. PTC–Platelet count.

Variables	*P*-value	Odds ratio (OR) for HMOD	CI 95%	
Age (> 50 years)	<0.001	3.774	2.174	6.552
Sex (female)	.072	.604	.349	1.046
Smoking status	.193	1.478	.820	2.664
Alcohol intake	.111	.629	.355	1.113
WC (>98 cm)	.331	1.284	.776	2.124
DM	.081	1.619	.942	2.782
HLP	.246	1.419	.785	2.565
ARBs	.023	2.095	1.109	3.956
CCBs	.778	.917	.503	1.674
Diuretics	.111	.629	.355	1.113
Statins	.876	.957	.554	1.654
PTC (<275 10^3/μl)	.008	2.055	1.211	3.485
ESR (>20 mm/h)	.024	1.887	1.087	3.277
Fibrinogen (>320 mg/dL)	<0.001	2.968	1.640	5.373
Uric acid (>7 mg/dL)	.049	1.788	1.003	3.186
Ferritin (>150 μg/L)	.383	1.231	.772	1.963

Omnibus test for coefficients (Chi^2): P< 0.05. Cox and Snell R-squared: 0.272. Nagelkerke's R-squared: 0.365. Sensitivity= 0.70. Specificity= 0.78.

WC−Waist circumference. ESR–Erythrocyte sedimentation rate. PTC–Platelet count.
